# Feasibility of basic transesophageal echocardiography in hemorrhagic shock: potential applications during resuscitative endovascular balloon occlusion of the aorta (REBOA)

**DOI:** 10.1186/s12947-018-0129-8

**Published:** 2018-07-16

**Authors:** William A. Teeter, Bianca M. Conti, Phil J. Wasicek, Jonathan J. Morrison, Dawn Parsell, Bryan Gamble, Melanie R. Hoehn, Thomas M. Scalea, Samuel M. Galvagno

**Affiliations:** 1 University of North Carolina, Raleigh, North Carolina USA; 2 Department of Anesthesiology, Division of Trauma Anesthesiology, University of Maryland School of Medicine, Baltimore, MD USA; 3 Department of Surgery, University of Maryland School of Medicine, Baltimore, MD USA; 4 Department of Surgery, Program in Trauma, University of Maryland School of Medicine, Baltimore, MD USA; 5 Department of Surgery, Walter Reed National Medical Center, United States Army, Bethesda, MD USA; 6 Department of Surgery, Emory University, Atlanta, GA USA; 7 University of Maryland, Program in Trauma, Baltimore, MD USA; 8 Department of Anesthesiology, Divisions of Critical Care Medicine and Trauma Anesthesiology, University of Maryland School of Medicine, Baltimore, MD USA

## Abstract

**Background:**

There are numerous studies in the cardiovascular literature that have employed transesophageal echocardiography (TEE) in swine models, but data regarding the use of basic TEE in swine models is limited. The primary aim of this study is to describe an echocardiographic method that can be used with relative ease to qualitatively assess cardiovascular function in a porcine hemorrhagic shock model using resuscitative endovascular balloon occlusion of the aorta (REBOA).

**Methods:**

Multiplane basic TEE exams were performed in 15 during an experimental hemorrhage model using REBOA. Cardiac anatomical structure and functional measurements were obtained. In a convenience sample (two animals from each group), advanced functional cardiovascular measurements were obtained before and after REBOA inflation for comparison with qualitative assessments.

**Results:**

Basic TEE exams were performed in 15 swine. Appropriate REBOA placement was confirmed using TEE in all animals and verified with fluoroscopy. Left ventricular volume was decreased in all animals, and left ventricular systolic function increased following REBOA inflation. Right ventricular systolic function and volume remained normal prior to and after hemorrhage and REBOA use. Mean ejection fraction (EF) decreased from 64% (S.D. 9.6) to 62.1 (S.D. 16.8) after hemorrhage and REBOA inflation (*p* = 0.76); fractional area of change (FAC) decreased from 49.8 (S.D. 9.0) to 48.5 (S.D. 13.6) after hemorrhage and REBOA inflation (*p* = 0.82).

**Conclusion:**

Basic TEE, which requires less training than advanced TEE, may be employed by laboratory investigators and practitioners across a wide spectrum of experimental and clinical settings.

## Background

The porcine model is commonly used in cardiovascular research related to experimental models of hemorrhagic shock [1, 2]. Swine are mammalian vertebrates with a cardiovascular system that is very similar to humans, and for this reason, have served as the dominant animal model for previous study designs published in the literature [3, 4]. For instance, with regard to vascular size, an 80–100 kg swine has an aortic diameter of 1.6–1.8 cm versus an aortic diameter of 1.6–2.4 cm in a similarly-sized human [1, 5]. However, several potentially significant differences exist. For example, porcine hearts are shaped more like a “valentine,” have different orientations for insertion of the superior and inferior vena cava, and have dissimilar atrial components [5]. Table 1 summarizes some anatomical differences between swine and humans.

**Table 1:** Major anatomical differences between porcine and human hearts

Features	Porcine Heart	Human Heart
Shape and Orientation	“Valentine shaped heart” which is oriented in line with the unguligrade stance of the pig	“Trapezoidal” shaped heart oriented in line with the orthograde posture of the human being
Presence of tubular appendage	Observed in the right atrium	Observed in the left atrium
Vena cava orientation	The superior and inferior vena cava opens into the right atrium at right angles to each other	The superior and inferior vena cava open into the right atrium in a straight line at 180 degrees
Pulmonary veins	Left atrium receives 2 pulmonary veins	Left atrium receives 4 pulmonary veins
Muscular moderator in right ventricle	Prominent and situated more superior in the right ventricle	Less prominent and situated more inferior in the right ventricle
Characteristic of Apical components	Contains coarse and broad trabeculations	Trabeculations are absent and apex is narrower
Aortic-Mitral fibrous continuity	Reduced as 2/3RD of aortic valve is supported by left ventricular musculature	Not reduced
Coronary Dominance	Left anterior descending coronary artery dominant	Right coronary dominant
Difference in right and left atrio-ventricular branches	Right atrio-ventricular branches are less developed than left-sided equivalents	No major differences exist between the right and left atrio-ventricular branches

Transesophageal echocardiography (TEE) is an invasive diagnostic modality that carries minimal risk but requires extensive knowledge about anatomy, physiology, and physics for appropriate application [6]. There are numerous studies in the cardiovascular literature that have employed TEE in swine models, but nearly all of these studies have dealt with cardiovascular physiology related to cardiac surgery [7], valvular abnormalities [8], myocardial infarction [9, 10], or cardiopulmonary resuscitation [11]. Data regarding the use of TEE in swine models is limited; one of the few papers describing the technique was published over 20 years ago [2], and baseline anatomical and hemodynamic parameters for TEE use in swine models have only recently been defined [1]. Both three-dimensional [8] and transthoracic ultrasound [12] in swine models have been described, but use of basic TEE in a swine model of hemorrhagic shock has not. Herein, we describe our experience using basic multiplane TEE to characterize hemodynamic changes in a large closed chest experimental swine model of non-compressible hemorrhage using resuscitative endovascular occlusion of the aorta (REBOA). The primary aim of this study is to describe an echocardiographic method that can be used with relative ease to qualitatively assess cardiovascular function in a porcine hemorrhagic shock model.

## Methods

### Animal model

The overall objective of this in vivo study was to characterize changes in arterial waveforms that occur during hemorrhage and uses Yorkshire swine (*Sus scrofa*) weighing between 70 and 90 kg. Once animals were induced into hemorrhagic shock, animals were subsequently enrolled into various substudies which are reported separately. This study was study was undertaken at a certified laboratory, following Institutional Animal Care and Use Committee (IACUC) approval. The study consisted of two phases: animal preparation and volume controlled hemorrhage.

General anesthesia was induced using intramuscular ketamine (10-15 mg/kg) and xylazine (1–2.2 mg/kg) followed by intravenous propofol and ketamine and maintained with isoflurane (minimum alveolar concentration [MAC] range 1–4%) by mask followed by tracheostomy intubation. Animals were ventilated using a volume-controlled mode of 6 cc/kg with an FiO2 of 40–100% to maintain SpO2 > 92%. The jugular veins were cannulated bilaterally to permit intravenous access and placement of a Swan-Ganz catheter. An open cystostomy was performed for urine drainage.

Hemorrhagic shock was induced by removing 40% of the animal’s blood volume over the course of 20 min from a femoral venous catheter. The first 20% of blood volume was removed over 7 min, and the remaining 20% of blood volume was removed over 13 min.

**Fig. 1:**
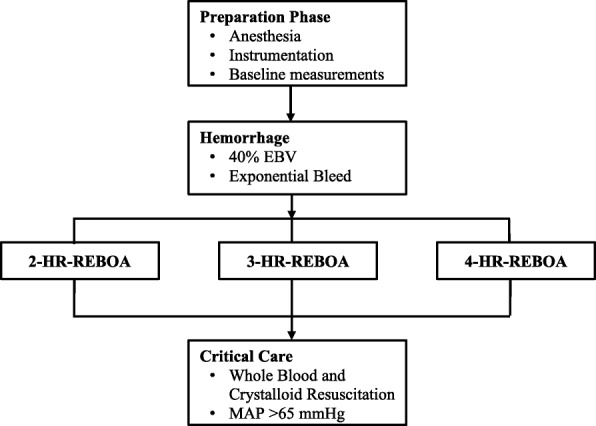
Summary of the three experimental hemorrhage REBOA models. EBV-estimated blood volume. MAP-mean arterial pressure. REBOA-resuscitative endovascular balloon occlusion of the aorta. The time interval indicates the amount of time the REBOA balloon was inflated in zone I of the animal

In order to prevent hemorrhage induced cardiac arrest, animals were given fluid boluses with 500 mL of lactated ringers, if in the judgement of the anesthetist, the animal was deteriorating to the verge of cardiac arrest. The animals were euthanized at the end of the protocol. The study design is summarized in Fig. 1.

### Resuscitative endovascular balloon occlusion of the aorta (REBOA)

**Fig. 2:**
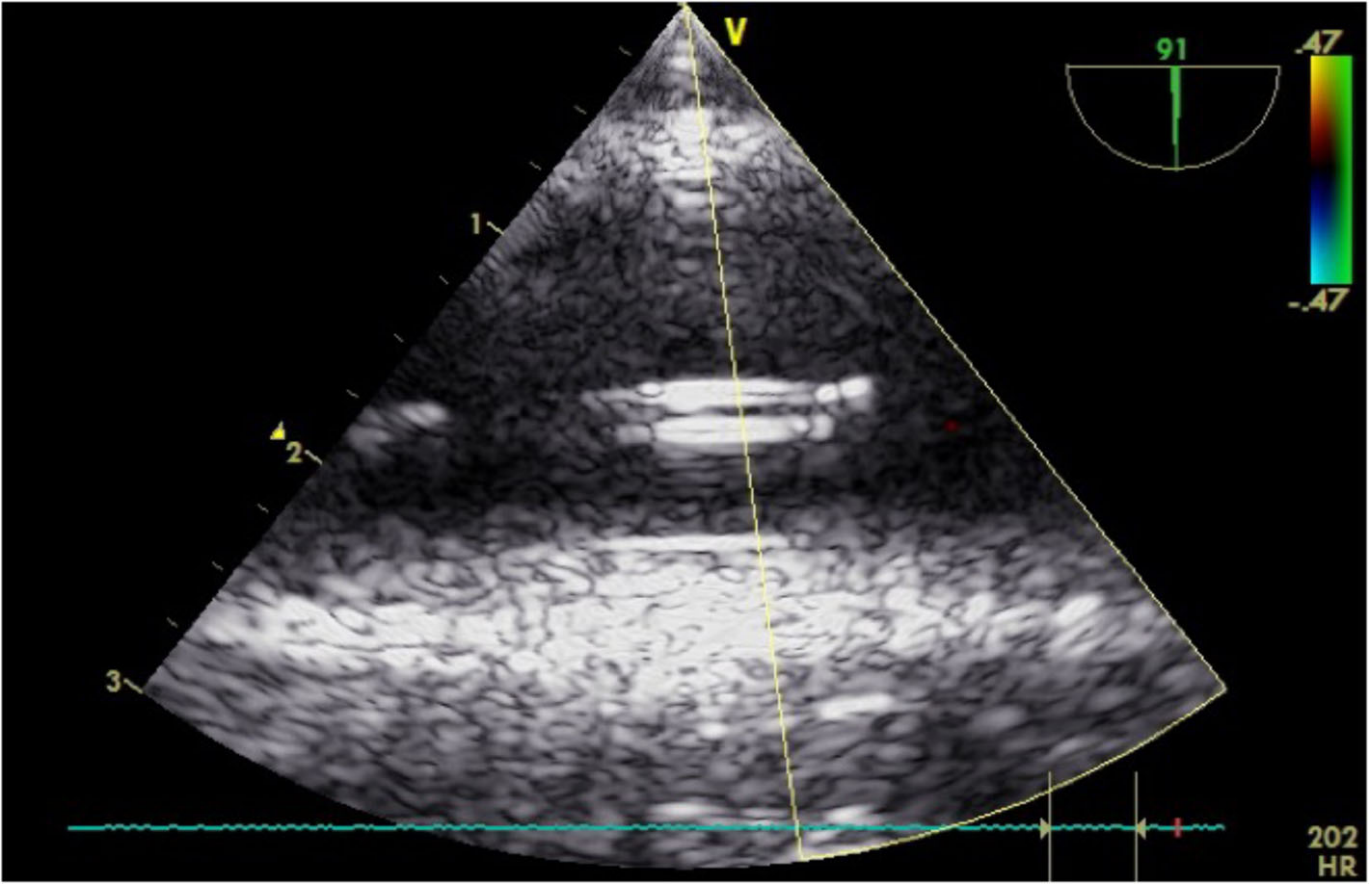
Midesophageal descending aortic long axis view. The hyperechoic structure located in the middle of the plane is the REBOA catheter

Resuscitative Endovascular Balloon Occlusion of the Aorta (REBOA) is a hemorrhage control technique which allows for the attenuation of hemorrhage by temporarily occluding the aorta in order to support blood pressure until definitive hemostasis can be achieved [13, 14]. There has been considerable interest in performing REBOA closer to the point of injury and for prolonged durations in order to lessen the effects of extended transport time prolonged field care [15, 16]. The physiologic effects of REBOA inflation for extended periods of time on the myocardium remains unknown. In this study, Zone 1 aortic occlusion was performed at the end of the hemorrhage protocol, with inflation of the balloon in the thoracic aorta (Fig. 2).

Aortic occlusion was confirmed using fluoroscopy.

### Multiplane TEE

Both baseline and post-hemorrhage multiplane TEE was performed under general anesthesia using a General Electrics LOGIQ e® ultrasound machine with a 6Tc-RS TEE probe (General Electrics Healthcare, Chicago, IL). TEE exams were performed by anesthesiologists certified by the National Board of Echocardiography; one investigator was certified at the basic TEE level (SG) and another at the advanced level (BC). For multiplane TEE, four maneuvers were used: [1] mechanical rotation of the variable plane phased array between 0° and 180°; [2] rotation of the shaft of the scope leftward and rightward; [3] advancement and withdrawal of the scope; and [4] tip anteflexion, retroflexion, and leftward and rightward flexion [2]. A basic TEE exam was performed using the 11 most relevant views recommended by the Consensus guidelines for basic perioperative TEE of the American Society of Echocardiography (ASE) and the Society of Cardiovascular Anesthesiologists (SCA) [6]. Specifically, the scope of practice for a basic TEE exam involves a limited application focused on intraoperative monitoring rather than specific diagnosis; a comprehensive exam, involving quantitative measurements is not within the scope of the basic perioperative TEE exam [6].

### Insertion of TEE probe

The TEE probe was inserted as previously described by Ren et al. [2] A disposable transducer sheath (100 cm long) was placed over the TEE probe with gel filling the inside and covering the tip of the probe. The distal portion of the probe was lubricated with gel and the probe was introduced while an assistant provided manual tongue elevation and retraction. With the swine in a supine position, the TEE scope was inserted blindly with the transducer array at 0° rotation by directing the tip into the posterior part of the pharynx, gently allowing the probe to flex passively, and advanced until images were obtained (approximately 45–60 cm from the incisors).

### Cardiac anatomical structure and function measurements

TEE images were recorded prior to experimental induction of bleeding and during occlusion with REBOA. The “iHeartScan™” Form was used to record qualitative and quantitative data (University of Melbourne, AUS). The iHeartScan™ is a limited echocardiography study that is qualitative and quantitative [17]. It is intended to be completed in approximately 10 min or 15 min for extended measurement. Components of the exam include qualitative assessments of ventricular volume in M-mode or 2D, systolic function (including both the right and left ventricle), left atrial filling pressure (assessed by observing interatrial sepal motion), valve assessment, presence of pericardial effusion, and estimation of overall hemodynamic state (i.e., vasodilated, primary systolic or diastolic failure, right ventricular failure) [17]. Extended aspects of the iHeartScan™ include calculation of ejection fraction, cardiac output, diastolic function (E/E’, E/A ratios, etc.), and valve measurements. Advanced hemodynamic calculations were made for selected animals. Measurements of chambers and great vessel diameters were obtained as recommended by the ASE/SCA Consensus guidelines [6]. Ejection fraction (EF) was calculated when using the Teichholz method [18, 19]. Stroke volume was calculated by multiplying the left ventricular outflow tract (LVOT) diameter x LVOT velocity time integral (VTI) [π (LVOT diameter/2)^2^ x LVOT VTI]; cardiac output was calculated by multiplying stroke volume x heart rate.

### Statistical analysis

Descriptive statistics were employed as appropriate according to the parametric or nonparametric nature of the data. Continuous data are described with a mean ± standard deviation. A *P* value of < 0.05 was considered statistically significant. Data were analyzed using the R package (version 3.1.1) for statistical computing (R Foundation for Statistical Computing, Vienna, Austria).

## Results

Basic TEE exams were performed in all 15 swine. The mean animal weight was 79.6 ± 5.5 kg. Heart rate significantly increased after REBOA inflation (76.8 [S.D. 21.3] vs. 151 [S.D. 47.1]; *p* < 0.001) with a corresponding significant increase in shock index, but mean arterial blood pressure did not change significantly. Qualitative basic TEE findings are summarized in Table 2.

**Table 2:** Summary of basic TEE findings and overall hemodynamic state during three experimental models (*n* = 5 animals in each phase)

Model	Timing	Shock Index	Pulmonary Artery Pressure	Mean Arterial Pressure	LV ventricular volume	RV ventricular volume	LV systolic function	RV systolic function	Hemodynamic state
2 h	Pre-bleed	0.63	37/27	93.5 (13.6)	Normal	Normal	Normal	Normal	Normal
	REBOA inflation	1.24*	47*/26	111.7 (29.1)	Hypovolemic	Normal	Increased	Normal	Empty
3 h	Pre-bleed	0.58	35/26	115.3 (13.2)	Normal	Normal	Normal	Normal	Normal
	REBOA inflation	0.96*	38/29	114.3 (14.8)	Hypovolemic	Normal	Increased	Normal	Empty; mild diastolic failure
4 h	Pre-bleed	0.56	33/24	116.6 (31.2)	Normal	Normal	Normal	Normal	Normal
	REBOA inflation	0.91*	38/27	100.3 (20.6)	Hypovolemic	Normal	Increased	Normal	Empty; mild diastolic failure

Basic TEE exams were performed pre-bleed and post-bleed during REBOA inflation. Findings are summarized qualitatively for all animals; hemodynamic findings are described with means and standard deviation. Shock index = heart rate / systolic blood pressure. *P < 0.01

With the exception of mean pulmonary artery systolic pressure in the 2-h experimental groups, no significant changes were observed in pulmonary artery pressures despite echocardiographic findings indicating hypovolemic shock. Similarly, there were no statistically significant differences in mean arterial blood pressure in any of the three study arms prior to and after REBOA inflation, despite experimental blood loss of 40%. In all animals, basic TEE qualitatively revealed increased LV function and hypovolemia as evidenced hyperdynamic physiology.

In a convenience sample (two animals from each group), advanced functional cardiovascular measurements were obtained before and after REBOA inflation for comparison with qualitative assessments. Mean ejection fraction (EF) decreased from 64% (S.D. 9.6) to 62.1 (S.D. 16.8) after hemorrhage and REBOA inflation (*p* = 0.76); fractional area of change (FAC) decreased from 49.8 (S.D. 9.0) to 48.5 (S.D. 13.6) after hemorrhage and REBOA inflation (*p* = 0.82). VTI and LVOT measurements were obtained in animals; mean cardiac output was 3.7 L/min prior to hemorrhage and REBOA inflation, and 7.6 L/min thereafter.

**Fig. 3:**
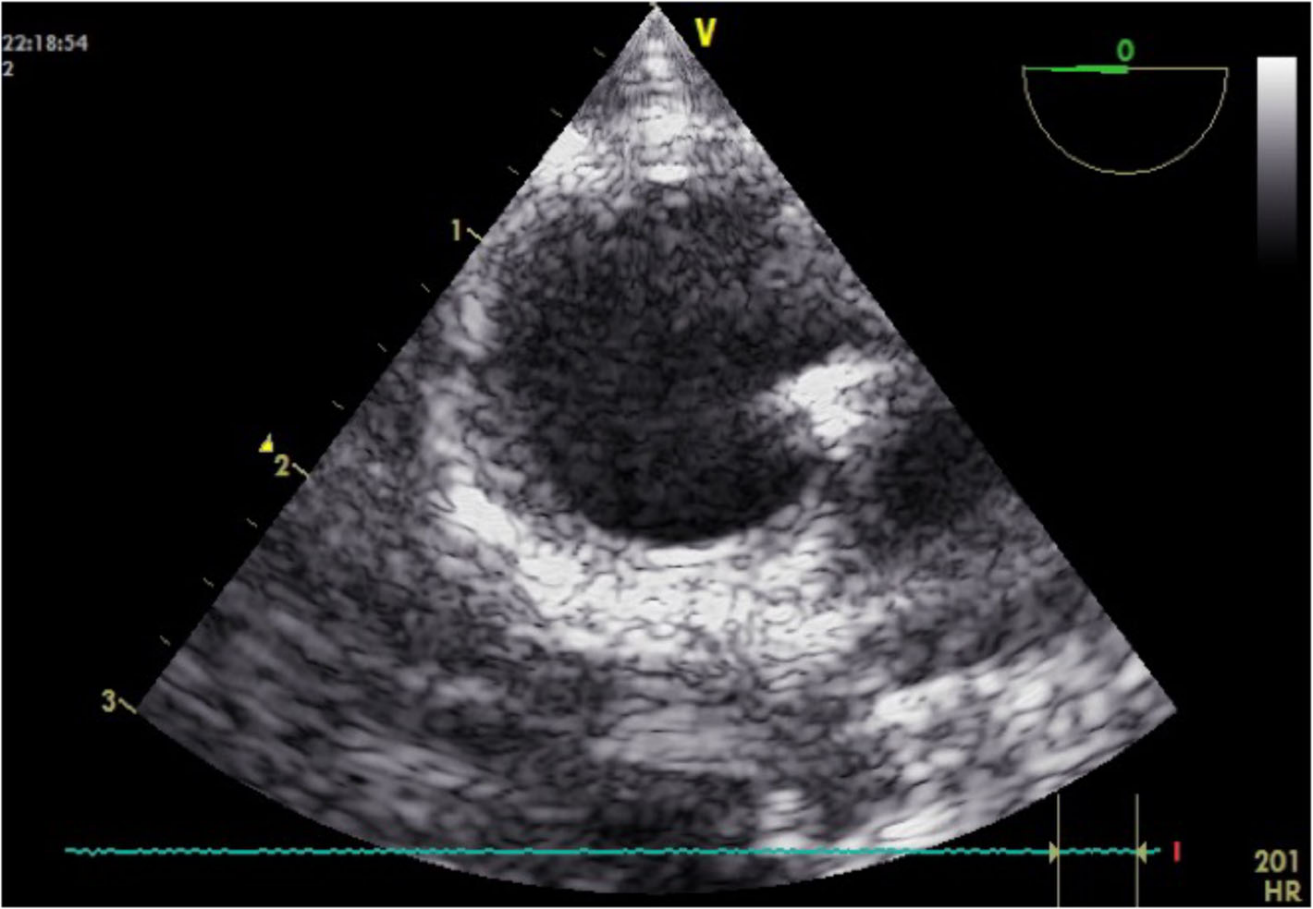
Midesophageal descending aortic short axis view. The hyperechoic structure located in the right lower quadrant of the aorta is the REBOA catheter

**Fig. 4:**
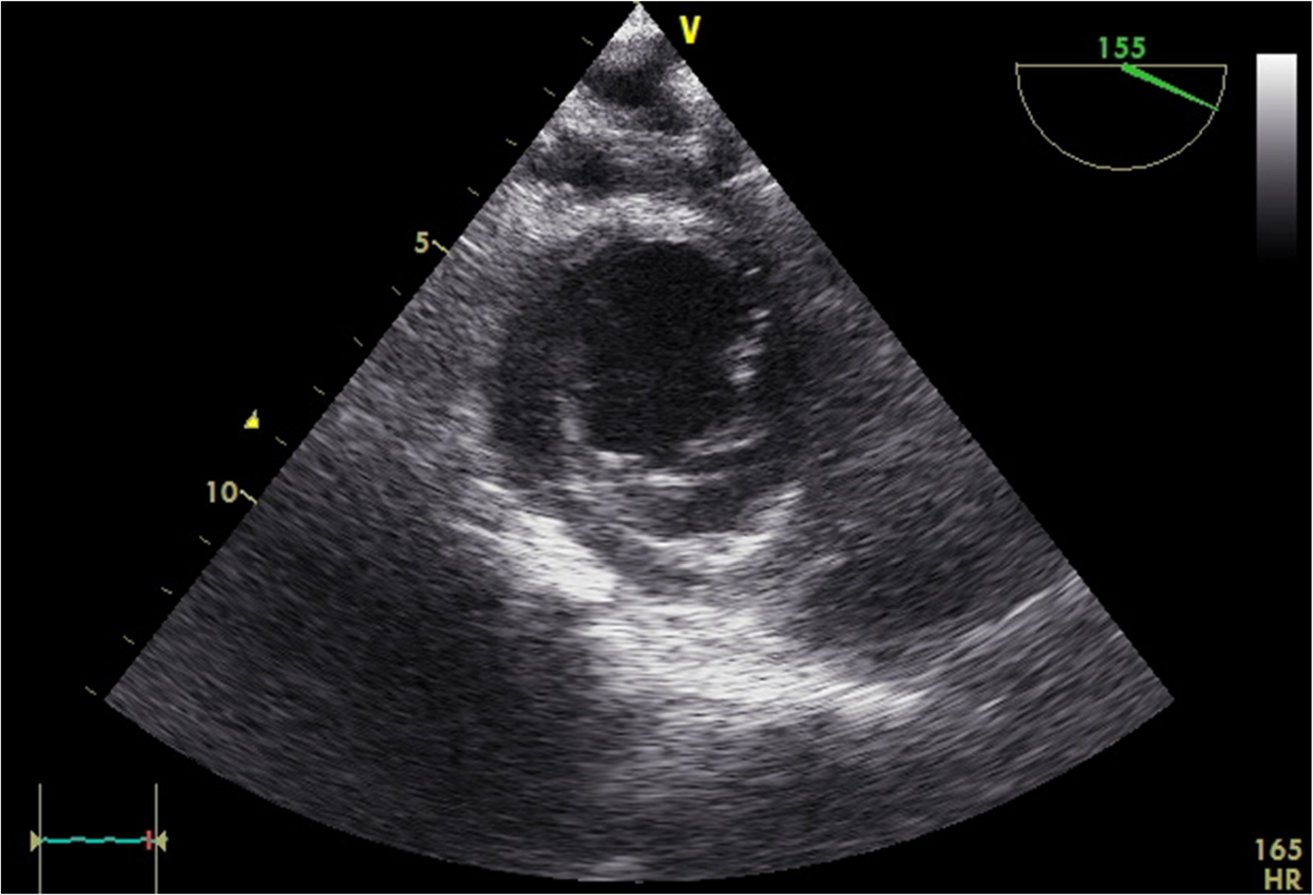
A representative transgastric short axis transesophageal view. Such a view is useful for assessing early regional wall abnormalities indicative of myocardial dysfunction and overall volume status

Midesophageal aortic long and short axis views were obtained to confirm correct REBOA catheter position (Figs. 2, 3, and 4). Catheter position was verified in all animals with basic TEE and confirmed with the use of fluoroscopy.

## Discussion

In a porcine closed chest experimental swine model of non-compressible hemorrhage using REBOA, use of basic multiplane TEE detected hemodynamic changes that could not be verified with the use of mean arterial pressure and pulmonary artery catheter data. With the increasing use of REBOA in both experimental settings and clinical settings, methods to rapidly and accurately assess hemodynamics are required. The use of basic TEE is of interest to laboratory investigators and clinicians for several reasons. First, the goal of basic TEE is intraoperative monitoring; while TEE is invasive, at the basic level, TEE can be used by a wide variety of scientists and clinicians to observe hemodynamic changes associated with aortic occlusion and hemorrhage. Second, basic TEE may reveal earlier evidence of hypovolemia despite relatively normal vital signs. Third, basic TEE may be helpful for localizing and confirming REBOA position. The primary goal of the basic TEE exam is intraoperative monitoring. The exam is not designed for practitioners to use the full diagnostic potential of TEE; hence, training requirements are considerably less than required for the advanced TEE exam [6]. Performance of basic TEE may be feasible in a wide variety of settings, including the laboratory, intensive care unit, and austere medical environments. Currently, “certification” is only available for licensed physicians who have passed an exam and demonstrated supervised performance of exams, but such requirements do not preclude performance of basic TEE exams in the laboratory by properly supervised investigators.

Limited application of TEE is potentially useful in both experimental and clinical settings where REBOA is employed for hemorrhage control because cardiac dysfunction is common in critical illness, but the effects on porcine and human hearts during REBOA are uncertain [20]. Basic TEE was feasible for all animals in our study. Clear evidence of hypovolemia was observed in all animals despite normal systemic and pulmonary blood pressure measurements. In animals with prolonged balloon inflation, evidence of early diastolic dysfunction was also observed, although in the subset of animals where EF and FAC were measured, no statistically significant differences were observed. These findings indicated a hyperdynamic cardiovascular state consistent with compensated shock. Such findings would be of great importance for both laboratory investigators and clinicians because further decreases in heart function would likely signify the upper limit of REBOA insertion before instituting definitive blood component resuscitation and surgical correction of hemorrhage.

An additional advantage of using basic TEE is for confirmation of proper REBOA location when fluoroscopic methods are not available. In previous studies, the subxiphoid view from a Focused Abdominal Sonogram for Trauma (FAST) was shown to reliably identify a central aortic guidewire, but in situations where the abdomen or chest is opened, such an exam may not be practicable [21]. In austere or limited resource settings when REBOA may be required as a temporizing measure for severe hemorrhage, fluoroscopy will likely be unavailable whereas a modern, compact TEE machine might be. In our study, a portable ultrasound machine weighing 11.5 lbs. (5.2 kg) was used. Moreover, use of TEE for both hemodynamic assessment and proper REBOA positioning obviates any exposure to radiation. In situations where REBOA has been employed for life-threatening hemorrhage, TEE has been used to confirm guidewire placement in the descending aorta [22]. Malpositioned REBOA, including inappropriate advancement into the ascending aorta, has potentially catastrophic consequences, including arrhythmia, carotid dissection, and coronary vasospasm [21]. Use of basic TEE is feasible with the advent of smaller, portable machines and probes, thus limiting radiation exposure and reducing the “footprint” associated with fluoroscopy.

There are several limitations to this work. Although the basic TEE exam requires less training and is easier to perform, advanced measurements, including functional measurement of stroke volume, cardiac output, stroke volume variation, and regional myocardial wall motion would likely be helpful to measure in future studies. Advanced calculations were obtained randomly in each experimental group for this study, but regular measurement of these parameters requires a more thorough exam performed by a practitioner with advanced TEE training. Basic TEE measurements were obtained prior to experimental hemorrhage and after REBOA inflation post-hemorrhage. Additional TEE exams conducted at regular intervals might have been more likely to reveal hemodynamic changes.

## Conclusions

Basic TEE is feasible in porcine hemorrhagic shock models involving REBOA. This method can be performed with relative ease and may assist with early detection of hypovolemia and confirmation of precise proximal REBOA deployment. Basic TEE requires less training than advanced TEE and may be employed by laboratory investigators and practitioners across a wide spectrum of experimental and clinical settings.

## Abbreviations

ASE: American Society of Echocardiography
AUS: Australia
EF: Ejection fraction
FAC: Fractional area of change
FAST: Focused abdominal sonogram for trauma
IACUC: Institutional animal care and use committee
LVOT: Left ventricular outflow tract
MAC: minimal alveolar concentration
REBOA: Resuscitative endovascular balloon occlusion of the aorta
S.D.: Standard deviation
SCA: Society of Cardiovascular Anesthesiologists
TEE: Transesophageal echocardiography
VTI: Velocity time integral
